# Global Gene Expression Profiling Reveals SPINK1 as a Potential Hepatocellular Carcinoma Marker

**DOI:** 10.1371/journal.pone.0059459

**Published:** 2013-03-18

**Authors:** Aileen Marshall, Margus Lukk, Claudia Kutter, Susan Davies, Graeme Alexander, Duncan T. Odom

**Affiliations:** 1 Cancer Research UK Cambridge Research Institute, Li Ka Shing Centre, Cambridge, United Kingdom; 2 Department of Histopathology, Addenbrooke’s Hospital, Cambridge, United Kingdom; 3 Cambridge Hepatobiliary Unit, Addenbrooke’s Hospital, Cambridge, United Kingdom; 4 Department of Oncology, Addenbrooke's Biomedical Campus, Hutchison-MRC Research Centre, Cambridge, United Kingdom; Institute of Hepatology, Foundation for Liver Research, United Kingdom

## Abstract

**Background:**

Liver cirrhosis is the most important risk factor for hepatocellular carcinoma (HCC) but the role of liver disease aetiology in cancer development remains under-explored. We investigated global gene expression profiles from HCC arising in different liver diseases to test whether HCC development is driven by expression of common or different genes, which could provide new diagnostic markers or therapeutic targets.

**Methodology and Principal Findings:**

Global gene expression profiling was performed for 4 normal (control) livers as well as 8 background liver and 7 HCC from 3 patients with hereditary haemochromatosis (HH) undergoing surgery. In order to investigate different disease phenotypes causing HCC, the data were compared with public microarray repositories for gene expression in normal liver, hepatitis C virus (HCV) cirrhosis, HCV-related HCC (HCV-HCC), hepatitis B virus (HBV) cirrhosis and HBV-related HCC (HBV-HCC). Principal component analysis and differential gene expression analysis were carried out using R Bioconductor. Liver disease-specific and shared gene lists were created and genes identified as highly expressed in hereditary haemochromatosis HCC (HH-HCC) were validated using quantitative RT-PCR. Selected genes were investigated further using immunohistochemistry in 86 HCC arising in liver disorders with varied aetiology. Using a 2-fold cut-off, 9 genes were highly expressed in all HCC, 11 in HH-HCC, 270 in HBV-HCC and 9 in HCV-HCC. Six genes identified by microarray as highly expressed in HH-HCC were confirmed by RT qPCR. Serine peptidase inhibitor, Kazal type 1 (SPINK1) mRNA was very highly expressed in HH-HCC (median fold change 2291, p = 0.0072) and was detected by immunohistochemistry in 91% of HH-HCC, 0% of HH-related cirrhotic or dysplastic nodules and 79% of mixed-aetiology HCC.

**Conclusion:**

HCC, arising from diverse backgrounds, uniformly over-express a small set of genes. SPINK1, a secretory trypsin inhibitor, demonstrated potential as a diagnostic HCC marker and should be evaluated in future studies.

## Introduction

Hepatocellular carcinoma (HCC) is the fifth most common cancer worldwide and lies third as a cause of death from cancer [1]. Once rare in Western countries, HCC now is the most rapidly growing cause of cancer deaths in the USA and UK [2], [3]. The prognosis for patients with HCC is poor; only 20% are eligible for curative surgery at presentation, with limited therapeutic options for the remainder. The inability to make a timely diagnosis and the limited efficacy of palliative treatments for HCC contribute to the poor outcome.

The population most at risk for HCC are those with cirrhosis; the highest risk, estimated at 3 to 8% per year, is associated with cirrhosis due to chronic hepatitis B virus (HBV) or hepatitis C virus (HCV) infection [4]–[6]. Liver diseases associated with intermediate risk include hereditary haemochromatosis (HH) [7]–[9], an inherited condition causing iron overload and iron deposition in the liver and other organs, non-alcoholic fatty liver disease [10], alcohol-related liver disease [11] and primary biliary cirrhosis [12], [13], while those with autoimmune liver disease probably have a lower risk [14]–[16].

Surveillance for HCC is recommended for patients with cirrhosis [17] but detection of a malignant nodule in a nodular cirrhotic liver is often challenging. Regenerative nodules and dysplastic nodules are difficult to distinguish from HCC on imaging criteria alone and are also common in cirrhotic liver. Biopsy confirms the diagnosis in many, but is impractical if the lesion is inaccessible percutaneously, or if patients have impaired blood clotting due to cirrhosis. Furthermore, HCC are heterogenous tumours often arising with dysplastic nodules and differentiating HCC from pre-malignant dysplastic nodules may not be possible using all available diagnostic tests, including histopathology [18].

Early diagnosis of HCC increases the likelihood that curative treatment can be offered [19]. The combination of ultrasound with cross-sectional computed tomography or magnetic resonance imaging is the best approach currently. For lesions smaller than 2 cm, the positive predictive value of radiology is 100%, but many small HCC do not have all the typical features and the negative predictive value is only 42% [17]. Serum α-fetoprotein (AFP) is the most commonly used circulating tumour marker, but has such low sensitivity and specificity that international guidelines no longer recommend using AFP when screening for HCC [17]. Other candidate serological tumour markers have been proposed, such as lens culinaris agglutinin reactive AFP (AFP-L3), des-γ-carboxy prothrombin (DCP), protein-induced vitamin K absence or antagonist II (PIVKA-II) and golgi protein 73, which have been used in some but not all clinical settings [17]. There remains great interest in finding biomarkers that would improve early diagnosis or provide prognostic information, but none as yet have entered routine clinical practice. The intent of our study was to identify markers that might be developed for clinical application using new genomics and bioinformatics tools.

One area that has been under-explored is the role of liver disease aetiology in driving HCC development. Liver diseases that pre-dispose to HCC development have several shared but also several distinct clinical and pathological features. Therefore, we hypothesised that a novel approach to integrate global gene expression data anchored on the cause of background liver disease might identify either shared genes or genes unique to those liver diseases and associated with HCC development. We found that HCC, arising from diverse backgrounds, over-expressed a small set of common genes but most over-expressed genes were unique to the liver disease in which HCC originated. We selected serine peptidase inhibitor, Kazal type 1 (SPINK1), a secretory trypsin inhibitor from the gene set over-expressed in haemochromatosis-related HCC and demonstrated its potential as a diagnostic marker in HCC.

## Results

### Collection and Analysis of Liver Cancer Data

**Figure 1:**
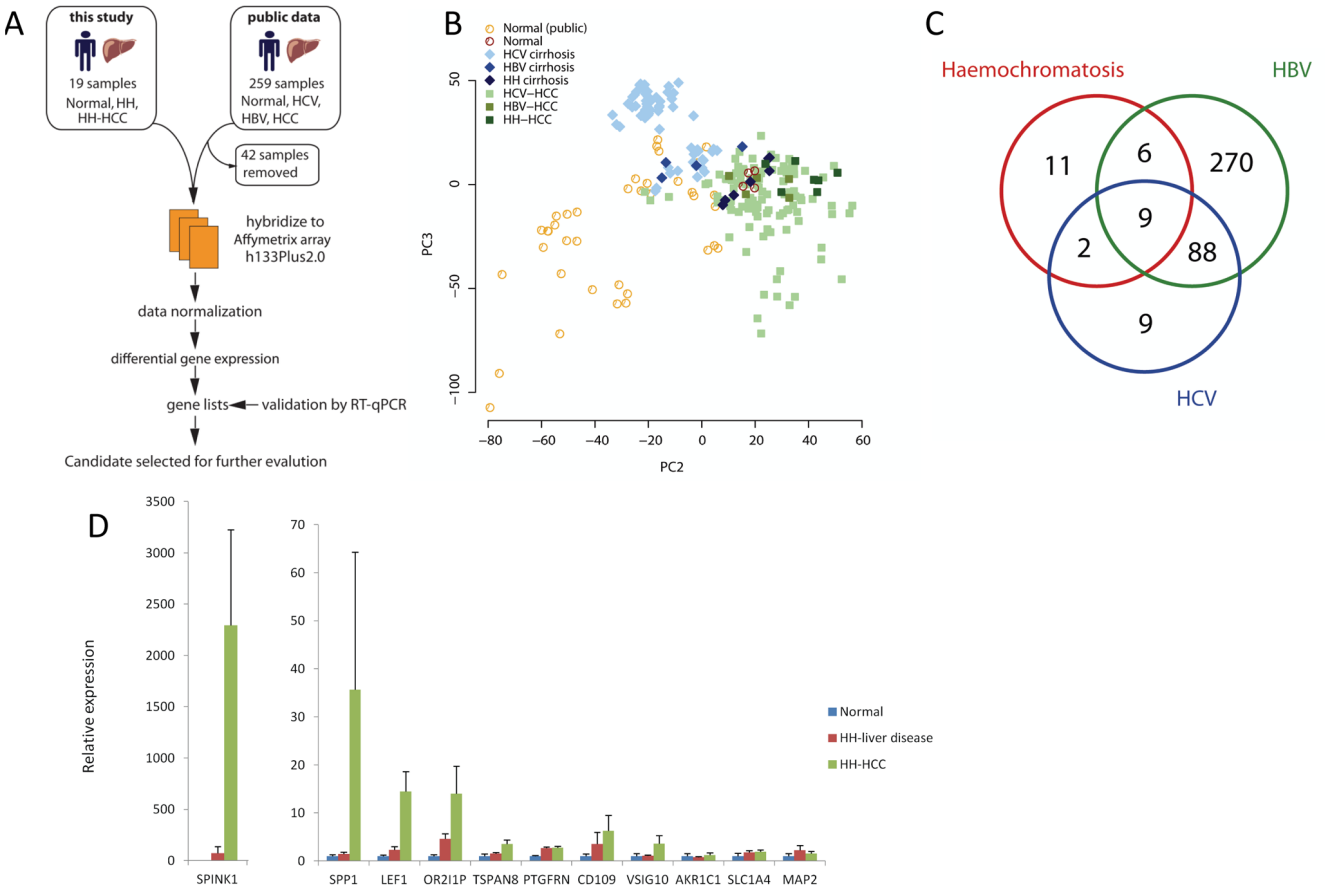
Global gene expression profiles in HCC, liver disease and normal liver reveal both unique and shared components. Candidate marker SPINK1 is highly up-regulated in HH-HCC. A) Flow diagram illustrating study outline. B) Principal component analysis of global gene expression profiles of normal liver, HCV liver disease, HCV-related HCC, HBV liver disease, HBV-related HCC, HH liver disease and HH-related HCC showing clustering between normal liver, liver disease and HCC samples. C) Venn diagram of differential gene expression, showing number of shared and unique differentially expressed genes between HCV-related HCC, HBV-related HCC and HH-related HCC all compared to normal liver and filtered for >2 fold cut-off. D) Reverse transcribed quantitative PCR for mRNA levels of selected genes identified by microarray analysis in normal liver, HH liver disease and HH-related HCC. Significant p-values for one-way anova: SPINK1 p = 0.0072, SPP1 p = 0.0354, LEF1 p = 0.001, OR2I1P p = 0.031, TSPAN8 p = 0.0181, PTGFRN p = 0.05. CD109, VSIG10, AKR1C1, SLC1A4 and MAP2 p = not significant.

A flow diagram outlining the study is shown in Figure 1A. The Affymetrix U133Plus2.0 array platform has been the most widely used microarray for profiling biological samples. We identified and curated 259 liver, liver disease and HCC gene expression profiles from public microarray data repositories ArrayExpress and Gene Expression Omnibus that had used the U133Plus2.0 microarray. We removed 42 samples from our analysis of the public data because they were from liver transplant recipients or dysplastic nodules. The remaining 217 samples fell into five groups: normal liver (n = 42), HCV liver disease (n = 59), HCV-HCC (n = 107), HBV liver disease (n = 4) and HBV-HCC (n = 5).

To allow cross-comparison with previously published data, gene expression profiles were generated using Affymetrix U133Plus2.0 for a set of samples obtained at Addenbrooke’s Hospital (see data accession in Materials and Methods). The experiments profiled samples from normal liver (4 patients) and both tumours and diseased background liver from 3 patients with hereditary haemochromatosis (HH) who underwent liver resection or liver transplantation for multiple HCC, which yielded background liver disease nodules (n = 8), HCC (n = 7), mixed HCC/cholangiocarcinoma (n = 1), dysplastic nodule (n = 1), regenerative nodules (n = 1) and necrotic nodule (n = 1). The clinical and pathological features for these cases are listed in Table 1. The mixed carcinoma, dysplastic, necrotic and regenerative nodules were not included in the analysis.

**Table 1:** Clinical and pathological data for cancers and liver nodules from patients with haemochromatosis.

	Patient 1	Patient 2	Patient 3
**Age (years)**	63	66	65
**Gender**	male	male	male
**Type of surgery**	liver transplant	liver resection	liver resection
**Total number and histology of** **liver lesions**	4 HCC	2 HCC	1 HCC and satellite nodules
	1 mixed CC/HCC		
	2 dysplastic nodules		
	2 regenerative nodules		
**Number of samples in microarray** **analysis**			
Background cirrhotic/fibrotic liver	4	2	2
Hepatocellular carcinoma	3	2	2
Mixed CC/HCC	1	0	0
Dysplastic nodule	1	0	0
Regenerative nodule	1	0	0
**Size of largest HCC (mm)**	21	60	95
**Serum α-fetoprotein (international** **units/ml, normal value <10)**	8	36	3733
**Background liver histology**	Cirrhosis and mild steatosis	Moderate – to – severe fibrosis,Grade 2/4 siderosis	Moderate fibrosis, Grade 2–3/4 siderosis

CC = cholangiocarcinoma.

Gene expression similarities of the samples were first explored by principal component analysis (PCA). As expected, the largest variation revealed by the first principal component was between in-house and public liver samples (data not shown). The second principal axis separated normal samples from HCC samples, leaving inflamed and cirrhotic samples between the two. The third principal axis captured variance within sample groups, as well as separating HCV-inflamed samples from the remainder. Visualisation of second and third axis together clearly distinguished three major, though partially overlapping, clusters: normal liver, background liver cirrhosis and HCC (Figure 1B).

### Comparison of Gene Expression from Different HCC Revealed Both Distinct and Common Signatures of Malignancy

Differential gene expression analysis was carried out comparing each disease group with normal liver. Lists of statistically significant genes were filtered for two-fold cut-off and categorised genes as unique to HH-HCC, shared between HH-HCC and HCV-HCC, shared between HH-HCC and HBV-HCC and shared between HH-HCC, HCV-HCC and HBV-HCC (all groups listed in Table 2). Figure 1C shows the number of these shared and unique genes in a Venn diagram. Only 9 differentially expressed genes were common to HH-HCC, HCV-HCC and HBV-HCC, listed at the foot of Table 2.

**Table 2:** Differentially expressed genes in HCC arising in different liver diseases compared to normal liver.

**Differentially expressed genes unique to HH-HCC**	
AKR1C1	aldoketoreductase family 1 member 1
CD109	CD109 molecule
CCNA2	cyclin A2
GPX2	glutathione peroxidase 2
MAP2	microtubule-associated protein 2
OR2I1P	olfactory receptor, family 2, subfamily I, member 1 pseudogene
PTGFRN	prostaglandin F2 receptor negative regulator
SPP1	secreted phosphoprotein 1
SLC1A4	solute carrier family 1 member 4
TOX3	TOX high mobility group box family member 3
VSIG10	V-set and immunoglobulin domain containing 10
**Differentially expressed genes shared between HH-HCC and HCV-HCC**	
SPINK1	serine peptidase inhibitor, Kazal type 1
TSPAN8	tetraspanin 8
**Differentially expressed genes shared between HH-HCC and HBV-HCC**	
TXNRD1	thioredoxin reductase 1
SOX9	SRY (sex determining region Y)-box 9
GABRE	gamma-aminobutyric acid (GABA) A receptor, epsilon
COL4A2	collagen, type IV, alpha 2
LRRC1	leucine rich repeat containing 1
NCRNA00152	non-protein coding RNA 152
**Differentially expressed genes shared between HH-HCC, HBV-HCC and HCV-HCC**	
RRM2	ribonucleotide reductase M2
SOX9	SRY (sex determining region Y)-box 9
CCL20	chemokine (C-C motif) ligand 20
GABBR1	gamma-aminobutyric acid (GABA) B receptor, 1
GPC3	glypican 3
SPP1	secreted phosphoprotein 1
CAP2	CAP, adenylate cyclase-associated protein, 2
LEF1	lymphoid enhancer-binding factor 1
MAP2	microtubule-associated protein 2

Genes are categorized as highly expressed in HH-HCC only, shared between HH-HCC and HCV-HCC, shared between HH-HCC and HBV-HCC or shared between all 3 groups.

Twenty-eight genes were highly expressed in HH-HCC, including those unique to HH-HCC or shared with another HCC group (Figure 1C, 28 corresponds to the total number of genes within the red circle and all listed in Table 2). We reviewed the individual plots of expression level for these 28 genes, comparing all liver disease, HCC and normal groups. We selected 11 genes with the greatest difference in expression level between HH-HCC and other sample groups and then validated these genes using reverse transcribed quantitative PCR (RTq-PCR). We found significant fold changes for SPINK1, LEF1, TSPAN8, SPP1, OR2I1P and PTGFRN comparing HH-HCC with HH-liver disease and normal liver (p<0.05, one-way Anova, Figure 1D). Primer sequences for RT qPCR are listed in Table S1.

Table S2 lists the 9 differentially expressed genes unique to HCV-HCC and Table S3 lists the 270 genes unique to HBV-HCC.

**Figure 2:**
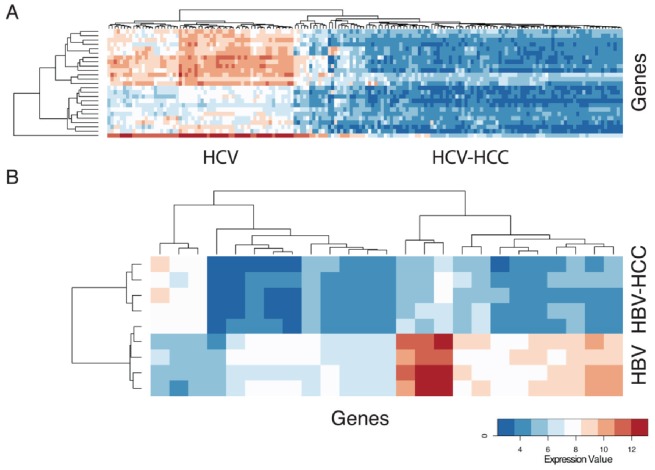
Gene expression profiles differentiate HCC from background liver cirrhosis. A) Heatmap of the 25 most significant genes with differential expression between HCV liver disease and HCV-related HCC. The gene names are listed in Table 3. B) Heatmap of the 25 most significant genes with differential expression between HBV liver disease and HBV-related HCC. The gene names are listed in Table 4.

In addition, comparison was made between HCV-related cirrhosis and HCV-HCC and HBV-related cirrhosis and HBV-HCC to identify genes unique to each disease that might be associated with progression to HCC. Figure 2A shows the heatmap of the 25 most significant genes with differential expression between HCV liver disease and HCV-HCC. These genes are listed in Table 3. Likewise, Figure 2B shows the heatmap of the 25 most significant genes with differential expression between HBV liver disease and HBV-HCC. These genes are listed in Table 4. Overall, more genes were significantly down-regulated in HCC compared to background viral hepatitis; transcriptional up-regulation was seen less often in HCC. The most significant gene ontology classifiers for all differentially expressed genes were various metabolic processes and immune responses (Table S4). Firstly, this might indicate loss of normal hepatocyte function in HCC due to de-differentiation of malignant cells. Secondly, many of the most significant genes down-regulated in HBV-HCC and HCV-HCC have immune function, including C-type lectins, ficolins and chemokine ligands, or are components of the extracellular matrix. These findings are in keeping with the prevailing view that a failure of anti-tumour immunity and altered tumour microenvironment are important factors allowing initiation and progression of HCC in cirrhosis [20], [21].

**Table 3:** The top 25 significant genes with differential expression, according to adjusted p-value, comparing HCV liver disease with HCV-HCC.

Gene symbol		log fold change	Adjusted p value
CLEC4G	C-type lectin domain family 4, member G	4.25	1.8×10^−63^
CLEC1B	C-type lectin domain family 1, member B	4.44	8.8×10^−58^
CLEC4M	C-type lectin domain family 4, member M	3.27	9.3×10^−57^
CLEC4M	C-type lectin domain family 4, member M	3.88	3.1×10^−56^
CRHBP	corticotropin releasing hormone binding protein	4.97	3.7×10^−55^
FCN2	ficolin (collagen/fibrinogen domain containing lectin) 2 (hucolin)	4.73	4.1×10^−54^
OIT3	oncoprotein induced transcript 3	4.60	5.9×10^−52^
MARCO	macrophage receptor with collagenous structure	3.17	2.8×10^−50^
CFP	complement factor properdin	2.46	2.2×10^−48^
LYVE1	lymphatic vessel endothelial hyaluronan receptor 1	3.54	2.5×10^−48^
RSPO3	R-spondin 3 homolog	2.72	4×10^−48^
FCN2	ficolin (collagen/fibrinogen domain containing lectin) 2 (hucolin)	2.67	4.9×10^−48^
STAB2	stabilin 2	2.03	6.6×10^−48^
CLDN10	claudin 10	3.12	1.7×10^−47^
CXCL14	chemokine (C-X-C motif) ligand 14	3.95	9.5×10^−47^
DPT	dermatopontin	2.47	1.3×10^−46^
GPM6A	glycoprotein M6A	3.03	1.8×10^−46^
FCN3	ficolin (collagen/fibrinogen domain containing) 3 (Hakata antigen)	4.56	3.8×10^−46^
CCBE1	collagen and calcium binding EGF domains 1	2.08	5.3×10^−46^
COLEC10	collectin sub-family member 10 (C-type lectin)	2.46	5.3×10^−46^
PLAC8	placenta-specific 8	3.93	4×10^−45^
LYVE1	lymphatic vessel endothelial hyaluronan receptor 1	2.83	9.7×10^−45^
TIMD4	T-cell immunoglobulin and mucin domain containing 4	3.29	2.1×10^−44^
HHIP	hedgehog interacting protein	2.78	3.2×10^−44^
CXCL14	chemokine (C-X-C motif) ligand 14	3.85	9.9×10^−44^

Genes may be represented on the microarray by more than one probeset, and fold change values are given for each individual probeset.

**Table 4:** The top 25 significant genes with differential expression, according to adjusted p-value, comparing HBV liver disease with HBV-HCC.

Gene symbol		log fold change	Adjusted p value
CRHBP	corticotropin releasing hormone binding protein	5.23	0.00006
GPM6A	glycoprotein M6A	4.06	0.00006
HAMP	hepcidin antimicrobial peptide	6.5	0.00006
RSPO3	R-spondin 3 homolog (Xenopus laevis)	2.7	0.00009
GPM6A	glycoprotein M6A	4.13	0.00012
CXCL14	chemokine (C-X-C motif) ligand 14	4.31	0.00019
CXCL14	chemokine (C-X-C motif) ligand 14	4.17	0.00033
HHIP	hedgehog interacting protein	3.64	0.00033
TMEM27	transmembrane protein 27	3.91	0.0004
IGF2	insulin-like growth factor 2 (somatomedin A)	5.15	0.00045
FCN3	ficolin (collagen/fibrinogen domain containing) 3 (Hakata antigen)	4.8	0.00066
CLEC1B	C-type lectin domain family 1, member B	3.96	0.00081
PRSS8	protease, serine, 8	2.37	0.00081
ADAMTS2	ADAM metallopeptidase with thrombospondin type 1 motif, 2	2.13	0.0011
C19orf77	Transmembrane protein C19orf77 Precursor	2.63	0.0011
WFDC1	WAP four-disulfide core domain 1	2.2	0.00134
PLAC8	placenta-specific 8	3.93	0.00134
ATAD2	ATPase family, AAA domain containing 2	−2.67	0.00178
P2RY12	purinergic receptor P2Y, G-protein coupled, 12	2.07	0.00207
COLEC10	collectin sub-family member 10 (C-type lectin)	2.47	0.00207
OIT3	oncoprotein induced transcript 3	4.89	0.00207
ECM1	extracellular matrix protein 1	2.35	0.00207
ATAD2	ATPase family, AAA domain containing 2	−2.61	0.00223
USP31	ubiquitin specific peptidase 31	−2.1	0.00226
NPY1R	neuropeptide Y receptor Y1	3.64	0.00240

Genes may be represented on the microarray by more than one probeset, and fold change values are given for each individual probeset.

### Specific SPINK1 Upregulation in HH-HCC

Because SPINK1 was by far the most upregulated gene in HH-HCC validated by RT qPCR, it was chosen for further investigation as a potential diagnostic marker in HCC. SPINK1 is the HUGO Gene Nomenclature Committee approved name for the gene originally identified as a trypsin inhibitor in bovine pancreas [22] and first described as a candidate tumour marker in ovarian cancer [23], [24], SPINK1 has historically been called tumor-associated trypsin inhibitor (TATI) and pancreatic secretory trypsin inhibitor (PSTI)). Physiologically, SPINK1 is secreted by pancreatic acinar cells and prevents trypsin-catalyzed premature activation of pro-enzymes within the pancreas and pancreatic duct. SPINK1 is aberrantly expressed in a number of different cancers [25].

**Figure 3:**
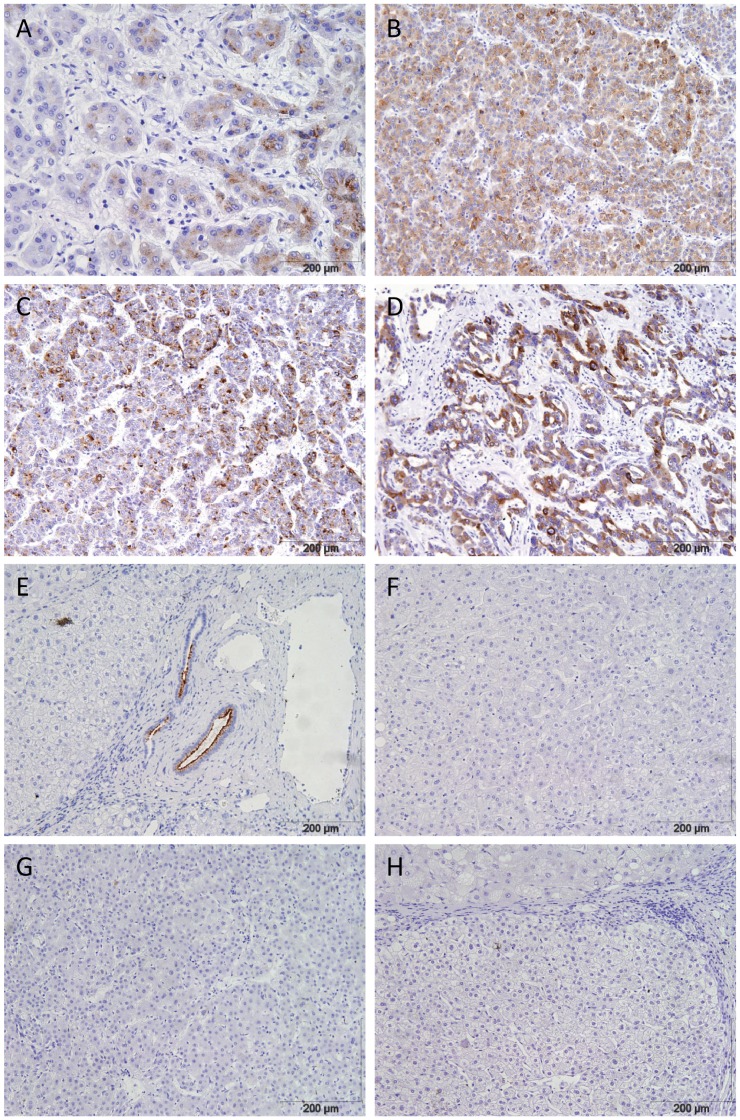
SPINK1 protein is detected in HH-HCC but not benign liver nodules. Immunohistochemistry for SPINK1 in HH liver disease, dysplastic and regenerative nodules and HCC. All images at 10× magnification. A–C) HH-related HCC from patients 1, 2 and 3. D) Mixed cholangiocellular carcinoma and HCC from patient 1. E) Background HH cirrhosis, showing positive SPINK1 expression in a large bile duct. F) Regenerative nodule. G) Dysplastic nodule. H) Diffuse small cell dysplasia.

We validated that SPINK1 was upregulated compared with both normal (p = 0.0283, Mann-Whitney U test) and HH background liver (p = 0.0281, Mann-Whitney U test, Figure 1D) using qPCR experiments. We confirmed the gene expression resulted in protein production by performing immunohistochemistry, and found that SPINK1 protein was detected in all HH-HCC (Figure 3 A–D). In HH-background liver, SPINK1 was detected on the luminal border of large bile ducts, but no hepatocyte expression was seen in HH-background liver (Figure 3E), regenerative nodules (Figure 3F), dysplastic nodules (Figure 3G), or diffuse small cell dysplasia (Figure 3H). This suggests that SPINK1 up-regulation is a late event in liver carcinogenesis and might represent a diagnostic target for established HCC.

Next, SPINK1 expression was assessed in tissue samples from a well-annotated clinical cohort (n = 86) of patients who had undergone liver transplantation for HCC between 1985 and 2004. Sixty-eight patients (79%) were male and the average age at transplant was 52.2 years. The background primary liver diseases were: HCV (n = 36), alcohol related liver disease (n = 12), HBV (n = 8), cryptogenic (n = 7), HH (n = 4), primary biliary cirrhosis (n = 4), autoimmune hepatitis with cirrhosis (n = 3), Wilson’s disease (n = 1), tyrosinaemia (n = 1), familial cirrhosis (n = 1), metabolic (n = 1), nodular regenerative hyperplasia (n = 1), non-cirrhotic (n = 1) and not recorded (n = 6).

The Milan criteria are used in most liver transplant units worldwide to minimise the rate of post-transplant HCC recurrence. According to the Milan criteria, liver transplantation can be offered to patients with one HCC smaller than 5 cm or up to 3 HCC smaller than 3 cm [26]. Addenbrooke’s Liver Transplant unit adopted the Milan criteria shortly after their publication in 1996. Overall, 32 patients had undergone transplantation prior to 1996 and 38 patients exceeded Milan criteria using histological measurements.

**Figure 4:**
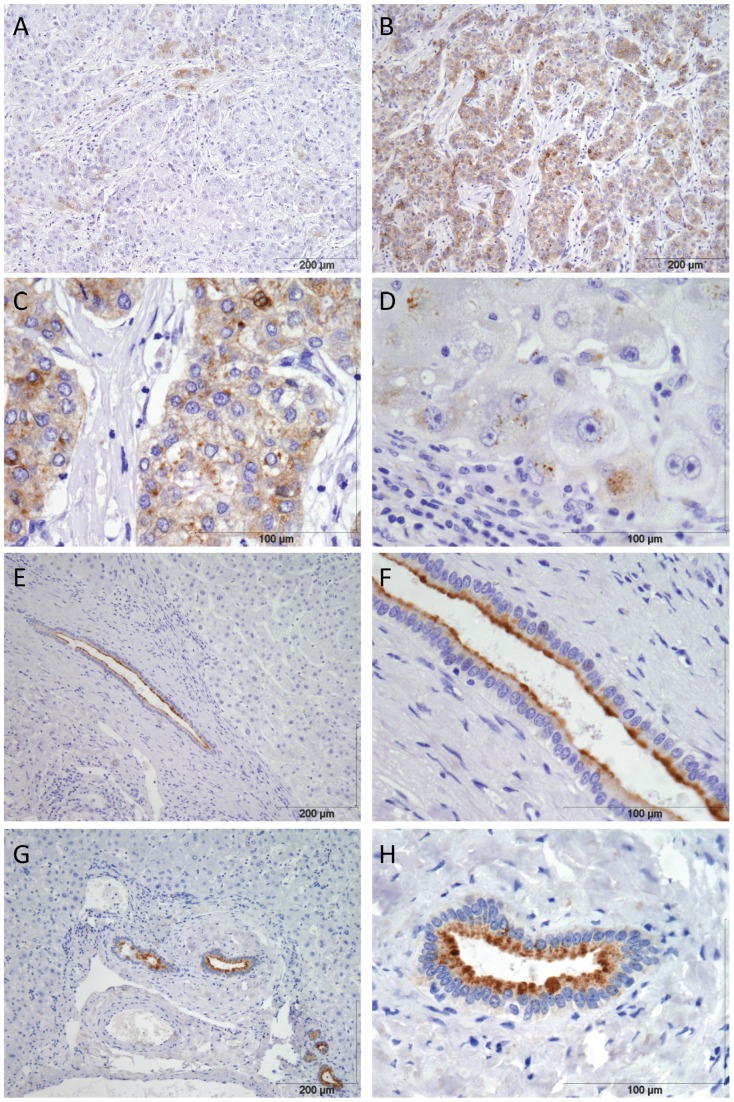
SPINK1 protein is detectable in HCC originating in different liver diseases but is only expressed in bile duct epithelium in liver cirrhosis and normal liver. Immunohistochemistry for SPINK1 A) Occasional positive tumour cells in HCC on background of HBV cirrhosis B) High frequency of SPINK1 expression in HCC on background of HCV cirrhosis at 10× magnification C) Same case as (B) at 40× magnification, showing cytoplasmic tumour cell expression D) Patchy periportal hepatocyte SPINK1 in advanced primary biliary cirrhosis 40× magnification. E) Background liver cirrhosis (HH) showing positive SPINK1 in large bile duct at10× magnification. F) Same case as (E) at 40× magnification showing SPINK1 localised to luminal surface of large bile duct. G) Normal liver showing positive SPINK1 in large bile duct at10× magnification. H) Same case as (G) at 40× magnification showing SPINK1 localised to luminal surface of large bile duct.

SPINK1-positive tumour cells were seen in 67 of the 86 (79%) HCC cases; the frequency of positive tumour cells ranged from occasional, dispersed cells (Figure 4A) to present in all tumour cells (Figure 4B and C). SPINK1-positive tumour cells were present in 79% of HCC overall, but were present in 91% of HH-HCC, 91% of ALD-HCC, 75% of HCV-HCC, 88% of HBV-HCC and 85% of cryptogenic-HCC. There was no evidence of a correlation between the proportion of SPINK1 positive tumour cells or intensity of staining and aetiology of liver disease.

The correlation between SPINK1 and clinical parameters is summarized in Table 5. The median age of patients with SPINK1-negative HCC was lower than patients with SPINK1-positive HCC (50.4 vs. 54.4 years, p = 0.03, Mann-Whitney U test). We tested whether SPINK1 was a prognostic marker by comparison with standard features known to be associated with outcome. However, we found no evidence of an association between SPINK1 expression and: (i) tumour size; (ii) vascular invasion; (iii) tumour grade, which was available from 67 cases, ranging from grade 1 (well-differentiated) to grade 3 (poorly differentiated). Finally, we compared SPINK1 status with treatment outcome. Tumour recurrence status was known for 75 patients surviving more than 6 months after transplantation; 32 of these patients exceeded Milan criteria according to histological measurement. Tumour recurrence had occurred in 18 out of 75 patients and there was no evidence of a difference in tumour recurrence comparing SPINK1 negative with SPINK1 positive HCC (22.2% vs. 24.6%, p = 1.00). In summary, SPINK1 appears to be a strong candidate as a diagnostic marker, but had no prognostic value.

**Table 5:** Clinical and histopathological data in 86 patients with HCC according to tumour cell SPINK1 status.

	SPINK1 negative HCC	SPINK1 positive HCC	p-value
Number	18	68	
Median age at transplant (inter-quartile range)	50.4 years (46.8–53.9)	54.4 years (49.7–61)	0.0295*
Gender (Male:Female)	14∶ 4	54∶ 14	1.00#
Median tumour size (IQR)	2.85 cm (2.43–5.25)	3 cm (2.08–4.63)	0.764*
Tumour grade (number grade1∶ 2∶ 3)	6∶ 8∶ 1	22∶ 23∶ 7	0.714†
Vascular invasion (Present : Absent)	8∶ 10	42∶ 26	0.282#
Within Milan criteria (Yes : No)	11∶ 7	37∶ 31	0.79#
Post transplant HCC recurrence (% with recurrence)	22.2	24.6	1.00#

IQR = interquartile range.

* Mann-Whitney U test,

# Fisher’s exact test for categorical variables,

† Chi squared test.

Any diagnostic marker needs to distinguish readily between non-tumour liver and HCC. SPINK1 expression in background non-tumour liver was localized to the luminal surface of large bile ducts in all cases (Figure 4E and F) which is compatible with the physiological function of SPINK1. Three samples of normal liver from patients who had undergone liver resection for colorectal cancer metastases also showed SPINK1 expression in the large bile duct epithelium (Figure 4G and H). These 3 normal livers and 17 of 18 background cirrhosis cases had no apparent transcription in hepatocytes. One case of primary biliary cirrhosis showed patchy periportal SPINK1 hepatocyte expression (Figure 4D). Periportal hepatocyte expression of biliary markers is well recognised in advanced PBC and other cholestatic diseases and we speculate that this explains positive hepatocyte SPINK1 in this case.

**Figure 5:**
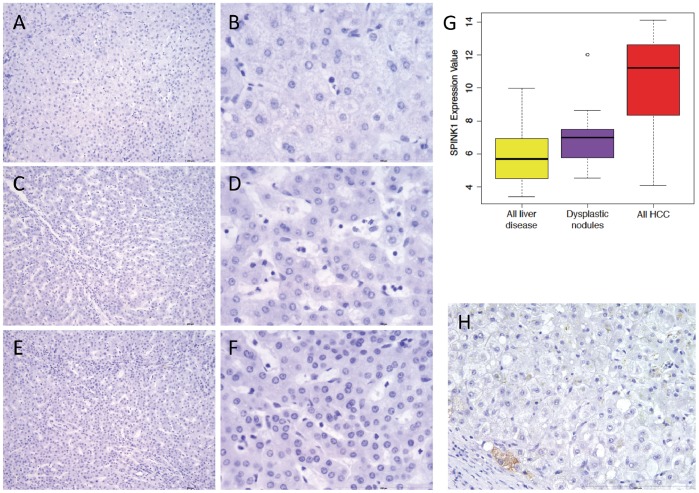
SPINK1 is not highly expressed in regenerative and dysplastic liver nodules. A–F and H: Immunohistochemistry for SPINK1 A) Macroregenerative nodule, 10× magnification B) Macroregenerative nodule, 40× magnification C) Low grade dysplastic nodule, 10× magnification D) Low grade dysplastic nodule, 10× magnification E) High grade dysplastic nodule 10× magnification F) High grade dysplastic nodule 10× magnification G) SPINK1 mRNA expression in public liver data, dysplastic nodules vs. all HCC, p = 3.8×10^−7^, dysplastic nodules vs. all liver disease, p = 0.04 H) SPINK1 immunohistochemistry showing rare positive cell in one low grade dysplastic nodule.

Two low grade dysplastic nodules and 3 macroregenerative nodules from HH patient 1 were negative for SPINK1 using immunohistochemistry. To investigate the expression of SPINK1 in regenerative and dysplastic liver nodules further, we first looked at the SPINK1 mRNA expression level in 17 dysplastic nodules arising in HCV liver disease in the public microarray data. SPINK1 mRNA expression was significantly higher in all HCC compared to the dysplastic nodules (p = 3.8×10^−7^, Figure 5G) and there was a modest increase in SPINK1 comparing dysplastic nodules with background liver diseases (p = 0.04, Figure 5G). Secondly, we sought SPINK1 expression in additional macroregenerative nodules (MRN), low grade dysplastic nodules (LGN) and high grade dysplastic nodules (HGN) from 5 patients who had undergone liver transplantation, giving a total of 8 MRN, 7 LGN and 3 HGN. The clinical and demographic details for these patients are in Table 6. All 8 MRN, 6 LGN and 2 HGN were negative for SPINK1 throughout (MRN, Figure 5A, B, LGN, Figure 5C, D and HGN Figure 5 E, F). One LGN and 1 HGN from the same individual, both with histological features of cholestasis and bile plugs were negative apart from very occasional positive cells at the nodule edge (Figure 5H). Overall, SPINK1 distinguished HCC from non-cancer liver disease and normal liver using standard immunohistochemistry.

**Table 6:** Clinical and pathological data for macroregenerative and dysplastic liver nodules.

	Age at transplant (years)	Gender	Background liver disease	Nodule types	SPINK1
**Patient 1**	63	Male	HH	MRN	Negative
				MRN	Negative
				MRN	Negative
				LGN	Negative
				LGN	Negative
**Patient 2**	68	Male	NASH	MRN	Negative
				LGN	Negative
				HGN	Negative
**Patient 3**	55	Male	HCV	MRN	Negative
				MRN	Negative
				LGN	Negative
**Patient 4**	66	Female	HCV	LGN	Negative
**Patient 5**	48	Male	HCV & ALD	LGN	Very low frequency
				HGN	Very low frequency
**Patient 6**	63	Male	HBV & ALD	MRN	Negative
				MRN	Negative
				LGN	Negative
				HGN	Negative

MRN = macroregenerative nodule LGN = low grade dysplastic nodules HGN = high grade dysplastic nodule HH = hereditary haemochromatosis, NASH = non-alcoholic steatohepatitis, HCV = hepatitis C virus, HBV = hepatitis B virus, ALD = alcohol-related liver disease.

## Discussion

Most patients with HCC have cancer too advanced at diagnosis for curative treatment, so improving early and accurate diagnosis is a priority. Attempts to identify gene expression signatures that predict prognosis have been hindered both by limited numbers and limited concordance between studies [27]. Unfortunately, no HCC studies have produced diagnostic targets that have been validated sufficiently to enter clinical practice. This highlights the heterogeneity of HCC and the difficulty in comparing gene expression data generated using different platforms. Given the importance of background liver disease to HCC risk, we hypothesised that part of the genetic heterogeneity of HCC might be explained by the underlying liver disease.

Most data available currently are derived from HCC caused by HBV or HCV infection. HCV-HCC is regarded primarily as inflammation driven. Inflammation is also important for HBV-HCC, but in addition, HBV DNA can integrate into host genome, thereby disrupting regulation of tumour suppressors or oncogenes [28]. Also, viral proteins including HCV core protein and HBV X, promote host cell malignant transformation [29], [30]. We collected comparable gene expression data from haemochromatosis as a distinct driver for liver cancer. In contrast to HBV and HCV cancers, the primary mechanism of injury in HH is oxidative damage; excess circulating ferrous iron (Fe^2+^) accumulates in hepatocytes, undergoes a Fenton reaction to yield Fe^3+^ and oxygen free radicals which then oxidise DNA bases, cellular proteins and lipids. Oxidised DNA bases, especially 8-oxoguanine, mismatch during DNA replication, leading to frequent G to C transversions.

By intersecting a large amount of data from HCC from different background liver diseases, we hoped to identify a set of potential diagnostic markers that would be specific for established liver cancers, but independent of aetiology. Conversely, genes specific to HCC originating on specific disease backgrounds may be useful for monitoring affected patients to improve early diagnosis of HCC. We addressed the discordance among studies and maximized the sample set available for our analysis by using the most widely-employed microarray platform, the Affymetrix U133Plus2.0. Our analysis revealed 9 genes that were strongly and reliably expressed in HCC from all 3 groups - HBV, HCV and haemochromatosis - whereas many more genes were differentially expressed in disease subsets (Figure 1B and Table 2).

The involvement of three of these 9 genes highly expressed in HH-HCC, HBV-HCC and HCV-HCC - glypican 3, osteopontin and microtubule-associated protein 2 - is well described. Both osteopontin and GPC 3 have been assessed as diagnostic HCC markers. Osteopontin may be useful as a circulating marker in HCV-related HCC [31]–[33] and although GPC3 is present in almost all HCC tissues, circulating GPC3 is not higher in patients with HCC compared to cirrhosis alone [34]–[36].

From the other genes differentially expressed in HCC, we chose to investigate SPINK1, nominally a pancreatic trypsin inhibitor, because of its very high fold change (median 2291) in mRNA expression between normal liver and HH-HCC. All HH-HCC in the 3 patients included in the microarray analysis were positive for SPINK1 by immunohistochemistry and, crucially, SPINK1 protein did not appear to be expressed in benign cirrhotic or macroregenerative nodules. Eight of 10 dysplastic nodules were negative for SPINK1 throughout, while the remaining 2 contained only a handful of positive cells localised to the nodule edge. Thus, it is a strong candidate to differentiate cancer from precancerous lesions in the liver. Indeed, two previous reports have demonstrated that SPINK1 is expressed in HCC; a small study of twenty viral hepatitis-related HCC found that all were positive [37]. A larger study of HBV and HCV-HCC found that 68% of HCC tissues were positive and SPINK1 expression was associated with portal vein invasion and recurrence following resection [38]. Functional studies using cell lines transfected with HBV or HCV suggest that SPINK1 is up-regulated by hepatitis viruses [39].

Initially reported as a candidate tumour marker in ovarian cancer in 1982 [23], [24], SPINK1 was originally named tumor-associated trypsin inhibitor (TATI) and pancreatic secretory trypsin inhibitor (PSTI), and is expressed in many other cancers, including breast [40] prostate [41]–[44], colon [45], [46], pancreatic/biliary [47]–[49], gastrointestinal [50] and renal [51], [52]. Functional studies in breast, prostate and liver cancer cell lines have suggested SPINK1 might inhibit apoptosis [39]–[41].

SPINK1 over-expression may promote invasion and metastasis of cancer cells through a number of potential mechanisms [53], [54]. The prostate cancer cell line 22Rv1 has an aggressive phenotype and highly expresses SPINK1. In this cell line, high SPINK1 expression increased cell proliferation and invasion both in vitro and in tumour xenografts [55]. Furthermore, SPINK1 shares structural similarity with epidermal growth factor (EGF), an important growth factor in hepatocellular and many other cancers [56]. SPINK1 can activate the EGF receptor and treatment of tumour xenograft-bearing mice with antibodies to SPINK1 or EGR receptor reduced tumour growth [55], suggesting SPINK is a potential therapeutic target.

Interleukin 6 (IL6) is an important cytokine produced during chronic hepatitis [57] and is known to increase SPINK1 expression in hepatoma cell lines [58] through an IL6 responsive element in the SPINK1 gene. Theoretically, higher levels of IL6 in chronic hepatitis and cirrhosis might promote HCC through increasing SPINK1. However, we did not detect hepatocyte SPINK1 protein expression in the vast majority of cirrhosis samples in this study, suggesting that other factors are also needed to allow SPINK1 expression in HCC cells.

SPINK1 is a secreted protein and is therefore a candidate circulating tumour marker. Detection of circulating SPINK1 protein or mRNA has been described in a number of cancers [37], [46], [47], [59]–[61]. Indeed, a previous study used SPINK1 in a larger panel of blood markers for hepatopancreatobiliary (HPB) cancer [47]. SPINK1 was not useful in distinguishing HPB cancer in this group, however, the cancers were predominantly pancreatic and cholangiocarcinoma and the control group predominantly gallstone disease. Therefore, there are no published data on serum SPINK1 comparing HCC and cirrhosis patients. A recent study reports on the development and comparison of SPINK1 enzyme-linked immunosorbent and time-resolved immunofluorometric assays [62]. Evaluation of circulating SPINK1 as a diagnostic marker specific for HCC is an important future area of research.

In our study, SPINK1-positive tumour cells were present in virtually every HCC case occurring in a background of haemochromatosis or ALD, it is in these cases where its use as a diagnostic marker would likely be most effective. Of note, the existing serum marker AFP was in the diagnostic range for HCC in only one of the three patients with HH-HCC in this study. HCC arising in other background liver diseases still showed strong prevalence for SPINK1 (typically >75%), so its effective and reliable clinical use would require other indicative markers.

### Limitations

Despite comparing data from only the most widely used platform, our total number of samples was in the low hundreds. In addition, the number of samples of HBV and HH were under-represented relative to HCV. There were no public data for HH-HCC to compare with our own data. Finally, gene expression data from HCC related to the most prevalent contributors to HCC progression in the West, namely, alcoholic liver disease or non-alcoholic fatty liver, have not been reported on the microarray platform we studied.

### Summary

This integrated analysis revealed SPINK1 as a potential diagnostic marker that was validated using a set of well-characterized samples from different liver diseases. Further prospective studies are needed to demonstrate the use of SPINK1 in the clinical setting.

## Materials and Methods

### Ethics Statement

All patients gave written informed consent for collection and use of their tissues and the study was approved by the Cambridge Local Research Ethics Committee.

### Public Liver Data

Liver related gene expression samples for Affymetrix U133Plus2.0 array platform were identified and downloaded from public microarray data repositories ArrayExpress [63] and Gene Expression Omnibus (GEO) [64] in November 2010. The sample meta-data was manually curated to ensure consistent annotation and non-liver tissue, liver cell lines, post-transplant liver and dysplastic nodules were excluded. The remaining 217 samples were grouped into 7 annotation groups.

### Data Normalisation and Principal Component Analysis

All raw gene expression measurements were normalised using Robust Multichip Average (RMA) from Affymetrix Bioconductor package [65]. The normalised data matrix was scaled in sample dimension by unit variance and zero means for principal component analysis (PCA). PCA was computed by prcomp function in R statistical computing environment.

### Differential Gene Expression Analysis

A separate list of differentially expressed genes was computed by comparison of each disease group to normal liver. Differential gene expression analysis was carried out by Bioconductor limma package [66]. Obtained p-values were corrected by Benjamini-Hochberg method [67]; the significance level alpha applied on corrected p-values was 0.05. Lists of significant genes were filtered for 2 fold average expression. Disease group specific probe set lists were obtained by exclusion of probe sets present in the lists of any other disease group. The Gene Ontology terms enrichment analysis for differentially expressed gene lists was carried out by topGO package from Bioconductor [68].

### Patients

Background liver and HCC tissues were collected from three patients with hereditary haemochromatosis (homozygous for C282Y HFE mutation) and single or multiple HCC. Two patients underwent liver resection and one underwent liver transplantation. Surgical specimens were evaluated immediately by a liver histopathologist; fresh samples from all liver lesions visible macroscopically were snap-frozen in liquid nitrogen and stored at −80°C. Final histological diagnosis for that lesion was determined by the matched formalin-fixed, paraffin-embedded block. Not all nodules were apparent macroscopically on the unfixed tissue so additional nodules were available as formalin-fixed, paraffin embedded sections. Four samples of normal liver were collected from patients undergoing liver resection for colorectal cancer liver metastasis. The samples were distant to the metastasis and showed normal histology in 2 patients and mild steatosis in 2 patients.

HCC from a cohort of patients, who had undergone liver transplantation between 1985 and 2004 at Addenbrooke’s Hospital, Cambridge, UK, were used to investigate SPINK1 expression in HCC from different liver diseases. Age, gender and liver disease were recorded prospectively in a database. The histopathology reports were used to obtain tumour size and vascular invasion status. Tumour grade was assessed in 67 cases by an expert liver histopathologist. Clinical records were reviewed to determine tumour recurrence and patients surviving less than six months after transplant were excluded.

Regenerative and dysplastic liver nodules from 5 patients who had undergone liver transplantation were used to investigate SPINK1 expression by immunohistochemistry. The patients were identified through the histopathology database and the histopathological diagnosis confirmed by an expert liver histopathologist.

### Preparation of Total RNA

RNA extraction was performed using Qiazol reagent then DNase treated (Turbo DNase, Ambion) and column purified (Qiagen RNeasy mini columns). RNA quality and quantity was measured using spectrophotometry at 260 and 280 nm and on a Bioanalyzer Eukaryote Total RNA Nano Series II chip (Agilent).

### Microarray Gene Expression Profile Analysis

Microarray experiments were performed by the Paterson Institute for Cancer Research microarray service. RNA was prepared as described above and processed using Affymetrix U133Plus2.0 arrays. RNA integrity number (RIN) values were between 6.5 and 9 for all samples. The labelling of the sample material, hybridisation and scanning of the microarrays was carried out according to Affymetrix standard protocols by Molecular Biology Core Facility in Paterson Institute for Cancer Research, University of Manchester.

### Quantitative RT-PCR

A total of 5 µg DNase treated, column purified RNA was used for cDNA synthesis (Invitrogen). Quantitative real-time polymerase chain reaction was performed using an Applied Biosystems 7900HT instrument. Primer sequences are listed in Table S1. The data were analysed using the ΔΔCt method with beta actin as the control gene.

### Immunohistochemistry

Formalin-fixed, paraffin embedded sections were processed for immunohistochemistry using a standard protocol. Heat-mediated antigen retrieval was performed by microwaving tissue sections for 10 minutes in 0.1 M citrate buffer, pH6. Hydrogen peroxide was used to block endogenous peroxidase; endogenous avidin and biotin were blocked using a Vector ABC kit. Mouse monoclonal anti-SPINK1 antibody (Novus Biologicals, H00006690-M01) was diluted 1∶500 and incubated with sections overnight at 4°C. Detection was performed using biotinylated donkey anti mouse secondary antibody, streptavidin-biotin-horseradish peroxidase complex and 3,3-diaminobenzidine to develop the stain.

### Statistical Analysis of Patient Data

Data were analysed using Graph Pad Prism 5 software. Gene expression fold changes by RT qPCR were assessed by one-way analysis of variance when comparing the increase from normal to cirrhosis to HCC and by Mann-Whitney U-test when comparing any two groups. For differences in patient demographics and tumour pathological data, continuous variables were assessed by Mann-Whitney U test and categorical variables by Fisher’s exact test.

This was a retrospective study and a post-hoc power calculation showed that the sample size of 58 SPINK1 positive HCC has 80% power to detect an increase in HCC recurrence rate from 23% (the observed rate for this cohort) to 39%.

### Data Accession

The combined in-house and public expression data is available from ArrayExpress repository under accession E-MTAB-950.

## Supporting Information

Table S1 Primer sequences used for qRT-PCR in normal, haemochromatosis background liver and haemochromatosis-related HCC.
(DOCX)

Table S2 Genes with >2-fold change in expression unique to HCV-related HCC compared to normal liver.
(DOCX)

Table S3 Genes with >2-fold change in expression unique to HBV-related HCC compared to normal liver.
(DOCX)

Table S4 Gene ontology analysis of differential gene expression, showing top 10 significantly enriched terms comparing HBV with HBV-HCC and comparing HCV with HCV-HCC.
(DOCX)
