# Supplementary material for: Global Gene Expression Profiling Reveals SPINK1 as a Potential Hepatocellular Carcinoma Marker
Source: PLoS One. 2013 Mar 18;8(3):e59459. doi: 10.1371/journal.pone.0059459 (PMC3601070; doi:10.1371/journal.pone.0059459)
Supplement: Table S1 — Primer sequences used for qRT-PCR in normal, haemochromatosis background liver and haemochromatosis-related HCC. (DOCX) [file pone.0059459.s001.docx]

| Gene symbol | Forward primer (5’-3’) | Reverse primer (5’-3’) |
| --- | --- | --- |
| VSIG10 | CCACATAGTTGGGCCAGAGT | TGTGGCTGAGGAACCTTACC |
| SLC1A4 | GGGCCATGTTATTCATGGAG | TGGGGAGAATAAACCTGCTG |
| AKR1C1 | AAGCCAGGGCTCAAGTACAA | GGCAACCAGAACAATGTCTTT |
| LEF1 | ACAGATCACCCCACCTCTTG | TGATGGGAAAACCTGGACAT |
| SPINK1 | TGTCTGTGGGACTGATGGAA | TCAACAATAAGGCCAGTCAGG |
| SPP1 | GCCGAGGTGATAGTGTGGTT | TGAGGTGATGTCCTCGTCTG |
| PTGFRN | GATGCACGCTCCTACCATTT | GGTTCTAGCCAGGTCACACC |
| CD109 | AGATGATCTCAATCATGTGG | ACACTGAAGCATCTTGGG |
| MAP2 | GGAGGAAGCAGCAGGTGGGGA | GTCTCCTGACACACCTCGCCG |
| OR2I1P | GCTACCTCCTGACCTTGACG | TAGTAGTGAAGCCCGCGTCT |
| TSPAN8 | TTGCTGTAGGTGCCATCATC | TCATTCACAATGCGATCAGAC |

**Table S1** Primer sequences used for qRT-PCR in normal, haemochromatosis background liver and haemochromatosis-related HCC.
