# Supplementary material for: Global Gene Expression Profiling Reveals SPINK1 as a Potential Hepatocellular Carcinoma Marker
Source: PLoS One. 2013 Mar 18;8(3):e59459. doi: 10.1371/journal.pone.0059459 (PMC3601070; doi:10.1371/journal.pone.0059459)
Supplement: Table S2 — Genes with >2-fold change in expression unique to HCV-related HCC compared to normal liver. (DOCX) [file pone.0059459.s002.docx]

**Table S2** Genes with > 2-fold change in expression unique to HCV-related HCC compared to normal liver. 16 probe sets mapped to ESTs and not annotated genes.

| Gene | | log fold change | adjusted p-value |
| --- | --- | --- | --- |
| MTR | 5-methyltetrahydrofolate-homocysteine methyltransferase | 2.13 | 1.81 x 10^-28^ |
| DDX60 | DEAD (Asp-Glu-Ala-Asp) box polypeptide 60 | 2.01 | 3.39 x 10^-18^ |
| FAM169A | family with sequence similarity 169, member A | 2.66 | 9.44 x 10^-17^ |
| FZD6 | frizzled homolog 6 | 2.00 | 8.68 x 10^-15^ |
| IFI27 | interferon, alpha-inducible protein 27 | 3.25 | 4.73 x 10^-19^ |
| ISG15 | ISG15 ubiquitin-like modifier | 2.03 | 5.12 x 10^-17^ |
| KRT23 | keratin 23 (histone deacetylase inducible) | 2.13 | 8.76 x 10^-10^ |
| LYZ | lysozyme | 2.18 | 1.72 x 10^-13^ |
| TOP2A | topoisomerase (DNA) II alpha 170kDa | 3.27 | 9.50 x 10^-23^ |
