# Supplementary material for: Global Gene Expression Profiling Reveals SPINK1 as a Potential Hepatocellular Carcinoma Marker
Source: PLoS One. 2013 Mar 18;8(3):e59459. doi: 10.1371/journal.pone.0059459 (PMC3601070; doi:10.1371/journal.pone.0059459)
Supplement: Table S3 — Genes with >2-fold change in expression unique to HBV-related HCC compared to normal liver. (DOCX) [file pone.0059459.s003.docx]

| Gene |  | log fold change | Adjusted p-value |
| --- | --- | --- | --- |
| CDKN2A | cyclin-dependent kinase inhibitor 2A | 2.40 | 2.76 x10^-17^ |
| TDRKH | tudor and KH domain containing | 2.82 | 2.56 x 10^-16^ |
| STC1 | stanniocalcin 1 | 2.98 | 4.28 x 10^-16^ |
| FAM122B | family with sequence similarity 122B | 2.04 | 6.44 x 10^-16^ |
| BUB1 | budding uninhibited by benzimidazoles 1 homolog | 2.33 | 7.92 X 10^-16^ |
| EML6 | echinoderm microtubule associated protein like 6 | 2.19 | 2.40 X 10^-15^ |
| SGOL2 | shugoshin-like 2 | 2.25 | 2.86 X 10^-15^ |
| SLC39A4 | Zinc transporter ZIP4 Precursor | 2.23 | 2.88 x 10E^-15^ |
| ASAP1 | ArfGAP with SH3 domain, ankyrin repeat and PH domain 1 | 2.06 | 4.71 X 10^-15^ |
| NUF2 | NUF2, NDC80 kinetochore complex component, homolog | 2.73 | 7.82 X 10^-15^ |
| NCAPG | non-SMC condensin I complex, subunit G | 2.42 | 1.86 X 10^-14^ |
| SLC7A11 | solute carrier family 7, member 11 | 2.79 | 4.77 X 10^-14^ |
| DLGAP5 | discs, large (Drosophila) homolog-associated protein 5 | 2.27 | 4.95 X 10^-14^ |
| OSBPL3 | oxysterol binding protein-like 3 | 2.09 | 7.48 X 10^-14^ |
| FANCD2 | Fanconi anemia, complementation group D2 | 2.08 | 2.42 X 10^-13^ |
| WDR67 | WD repeat domain 67 | 2.26 | 3.23 X 10^-13^ |
| STK39 | serine threonine kinase 39 | 3.23 | 4.10 X 10^-13^ |
| CENPK | centromere protein K | 2.22 | 8.32 X 10^-13^ |
| GJC1 | gap junction protein, gamma 1, 45kDa | 2.04 | 9.05 X 10^-13^ |
| RNF157 | ring finger protein 157 | 2.93 | 1.41 X 10^-12^ |
| BUB1B | budding uninhibited by benzimidazoles 1 homolog beta | 2.76 | 1.56 X 10^-12^ |
| UBAP2L | ubiquitin associated protein 2-like | 2.31 | 2.52 X 10^-12^ |
| TMEM98 | transmembrane protein 98 | 2.34 | 2.71 X 10^-12^ |
| DFNB31 | deafness, autosomal recessive 31 | 2.04 | 7.13 X 10^-12^ |
| C12orf75 |  | 2.31 | 1.66 X 10^-11^ |
| PTK2 | Protein tyrosine kinase 2 | 2.46 | 2.24 X 10^-11^ |
| HIST1H2BG | histone cluster 1, H2bg | 2.47 | 2.26 X 10^-11^ |
| ITGA6 | integrin, alpha 6 | 2.43 | 2.53 X 10^-11^ |
| ZIC2 | Zic family member 2 (odd-paired homolog, Drosophila) | 3.09 | 3.08 X 10^-11^ |
| C8orf33 | UPF0488 protein C8orf33 | 2.08 | 3.15 X 10^-11^ |
| BIRC5 | baculoviral IAP repeat-containing 5 | 2.27 | 4.04 X 10^-11^ |
| POGK | pogo transposable element with KRAB domain | 2.40 | 4.52 X 10^-11^ |
| CDC20 | cell division cycle 20 homolog | 2.31 | 5.31 X 10^-11^ |
| CDKN2B | cyclin-dependent kinase inhibitor 2B (p15, inhibits CDK4) | 2.41 | 7.41 X 10^-11^ |
| TRIM16 | tripartite motif-containing 16 | 3.43 | 1.17 X 10^-10^ |
| VASH2 | vasohibin 2 | 2.09 | 1.39 X 10^-10^ |
| H2AFX | H2A histone family, member X | 2.13 | 1.88 X 10^-10^ |
| CKAP2 | cytoskeleton associated protein 2 | 2.36 | 2.06 X 10^-10^ |
| FERMT1 | fermitin family member 1 | 2.12 | 2.08 X 10^-10^ |
| SF3B4 | splicing factor 3b, subunit 4, 49kDa | 2.17 | 5.71 X 10^-10^ |
| COL4A2 | collagen, type IV, alpha 2 | 2.05 | 6.22 X 10^-10^ |
| WDYHV1 | WDYHV motif containing 1 | 2.79 | 7.55 X 10^-10^ |
| NCRNA00094 | non-protein coding RNA 94 | 2.18 | 1.08 X 10^-09^ |
| TNFRSF21 | tumor necrosis factor receptor superfamily, member 21 | 2.19 | 1.21 X 10^-09^ |
| COL4A1 | collagen, type IV, alpha 1 | 2.54 | 1.25 X 10^-09^ |
| CDK1 | cyclin-dependent kinase 1 | 2.32 | 1.84 X 10^-09^ |
| UBR5 | ubiquitin protein ligase E3 component n-recognin 5 | 2.15 | 1.85 X 10^-09^ |
| TUFT1 | tuftelin 1 | 2.90 | 1.90 X 10^-09^ |
| CCL15 | chemokine (C-C motif) ligand 15 | 2.22 | 2.10 X 10^-09^ |
| G6PD | glucose-6-phosphate dehydrogenase | 3.08 | 2.13 X 10^-09^ |
| OVOS2 | Ovostatin homolog 2 Precursor | 2.10 | 2.68 X 10^-09^ |
| CDCA7 | cell division cycle associated 7 | 2.70 | 3.15 X 10^-09^ |
| BOP1 | block of proliferation 1 | 2.41 | 3.47 X 10^-09^ |
| SQSTM1 | sequestosome 1 | 2.38 | 3.86 X 10^-09^ |
| DTL | denticleless homolog (Drosophila) | 2.17 | 4.07 X 10^-09^ |
| NEDD4L | neural precursor cell expressed, developmentally down-regulated 4-like | 2.23 | 4.21 X 10^-09^ |
| SPTAN1 | spectrin, alpha, non-erythrocytic 1 (alpha-fodrin) | 2.09 | 4.21 X 10^-09^ |
| IGF2BP3 | insulin-like growth factor 2 mRNA binding protein 3 | 3.23 | 5.06 X 10^-09^ |
| CENPF | centromere protein F, 350/400kDa (mitosin) | 2.18 | 5.06 X 10^-09^ |
| CRNDE | colorectal neoplasia differentially expressed | 2.18 | 5.09 X 10^-09^ |
| SKAP2 | src kinase associated phosphoprotein 2 | 2.20 | 7.09 X 10^-09^ |
| HOXA3 | homeobox A3 | 2.65 | 8.56 X 10^-09^ |
| ASAP1 | ArfGAP with SH3 domain, ankyrin repeat and PH domain 1 | 2.19 | 8.79 X 10^-09^ |
| LOC389384 | Ankyrin repeat domain 57 pseudogene | 2.06 | 9.09 X 10^-09^ |
| KIF20A | kinesin family member 20A | 2.16 | 9.27 X 10^-09^ |
| TRRAP | transformation/transcription domain-associated protein | 2.04 | 1.23 X 10^-08^ |
| LRP12 | low density lipoprotein receptor-related protein 12 | 2.26 | 1.43 X 10^-08^ |
| TRIP13 | thyroid hormone receptor interactor 13 | 2.01 | 1.75 X 10^-08^ |
| HOXA13 | homeobox A13 | 2.12 | 2.33 X 10^-08^ |
| SMARCC1 | SWI/SNF related, matrix associated, actin dependent regulator of chromatin, subfamily c, member 1 | 2.35 | 2.66 X 10^-08^ |
| SOD2 | superoxide dismutase 2, mitochondrial | 3.41 | 3.52 X 10^-08^ |
| EPPK1 | Epiplakin 1 | 2.90 | 3.62 X 10^-08^ |
| EIF2C2 | Eukaryotic translation initiation factor 2C, 2 | 2.08 | 3.76 X 10^-08^ |
| ANKRD27 | ankyrin repeat domain 27 (VPS9 domain) | 2.23 | 4.28 X 10^-08^ |
| RRAGD | Ras-related GTP binding D | 2.80 | 4.44 X 10^-08^ |
| SACS | spastic ataxia of Charlevoix-Saguenay (sacsin) | 2.47 | 4.92 X 10^-08^ |
| EBF1 | early B-cell factor 1 | 2.45 | 5.80 X 10^-08^ |
| CHD7 | chromodomain helicase DNA binding protein 7 | 2.51 | 6.94 X 10^-08^ |
| DLG5 | discs, large homolog 5 (Drosophila) | 2.42 | 7.17 X 10^-08^ |
| BCAT1 | branched chain amino-acid transaminase 1, cytosolic | 3.03 | 8.06 X 10^-08^ |
| ASAP2 | ArfGAP with SH3 domain, ankyrin repeat and PH domain 2 | 2.94 | 8.27 X 10^-08^ |
| ENAH | enabled homolog (Drosophila) | 2.35 | 9.30 X 10^-08^ |
| GREB1 | growth regulation by estrogen in breast cancer 1 | 2.76 | 9.46 X 10^-08^ |
| PRCC | papillary renal cell carcinoma (translocation-associated) | 2.12 | 1.03 X 10^-07^ |
| HIST2H2BE | histone cluster 2, H2be | 2.79 | 1.40 X 10^-07^ |
| TM4SF1 | transmembrane 4 L six family member 1 | 2.57 | 1.65 X 10^-07^ |
| SESTD1 | SEC14 and spectrin domains 1 | 2.14 | 1.82 X 10^-07^ |
| SGK223 | Homolog of rat pragma of rnd2 | 2.29 | 2.19 X 10^-07^ |
| CENPJ | centromere protein J | 2.10 | 2.57 X 10^-07^ |
| LAMC1 | laminin, gamma 1 (formerly LAMB2) | 2.28 | 2.78 X 10^-07^ |
| NRCAM | neuronal cell adhesion molecule | 3.33 | 2.90 X 10^-07^ |
| SERPINI1 | serpin peptidase inhibitor, clade I (neuroserpin), member 1 | 2.03 | 2.94 X 10^-07^ |
| SLC39A10 | solute carrier family 39 (zinc transporter), member 10 | 2.45 | 3.19 X 10^-07^ |
| TMEM64 | transmembrane protein 64 | 2.24 | 3.56 X 10^-07^ |
| GOLGA8A | golgin A8 family, member A | 2.37 | 3.72 X 10^-07^ |
| KIF4A | kinesin family member 4A | 2.05 | 3.95 X 10^-07^ |
| TMCC1 | transmembrane and coiled-coil domain family 1 | 2.15 | 4.72 X 10^-07^ |
| KDM3B | lysine (K)-specific demethylase 3B | 2.03 | 5.01 X 10^-07^ |
| NBPF8 | neuroblastoma breakpoint family, member 8 | 2.40 | 5.15 X 10^-07^ |
| COX7B2 | cytochrome c oxidase subunit VIIb2 | 2.60 | 5.21 X 10^-07^ |
| PAGE4 | P antigen family, member 4 (prostate associated) | 2.30 | 5.82 X 10^-07^ |
| PTPRG | protein tyrosine phosphatase, receptor type, G | 2.68 | 5.95 X 10^-07^ |
| ADRBK2 | Adrenergic beta receptor kinase 2 | 2.79 | 6.51 X 10^-07^ |
| FAM83D | family with sequence similarity 83, member D | 2.11 | 6.71 X 10^-07^ |
| IPW | Imprinted in Prader-Willi (non-coding) | 2.01 | 8.90 X 10^-07^ |
| AXIN2 | axin 2 | 2.07 | 9.01 X 10^-07^ |
| GDAP1 | ganglioside-induced differentiation-associated protein 1 | 2.10 | 9.22 X 10^-07^ |
| POLR3C | polymerase (RNA) III (DNA directed) polypeptide C | 2.33 | 1.00 X 10^-06^ |
| FGF13 | fibroblast growth factor 13 | 2.67 | 1.04 X 10^-06^ |
| KIF2A | kinesin heavy chain member 2A | 2.45 | 1.11 X 10^-06^ |
| UBE2S | ubiquitin-conjugating enzyme E2S | 2.25 | 1.16 X 10^-06^ |
| GPR158 | G protein-coupled receptor 158 | 2.01 | 1.18 X 10^-06^ |
| ATP6V1C1 | ATPase, H+ transporting, lysosomal 42kDa, V1 subunit C1 | 2.07 | 1.22 X 10^-06^ |
| MTHFD1L | methylenetetrahydrofolate dehydrogenase (NADP+ dependent) 1-like | 2.20 | 1.24 X 10^-06^ |
| PEA15 | phosphoprotein enriched in astrocytes 15 | 2.05 | 1.44 X 10^-06^ |
| YWHAZ | tyrosine 3-monooxygenase/tryptophan 5-monooxygenase activation protein, zeta polypeptide | 2.50 | 1.63 X 10^-06^ |
| NEU1 | sialidase 1 (lysosomal sialidase) | 2.07 | 1.88 X 10^-06^ |
| CD109 | CD109 molecule | 2.08 | 1.89 X 10^-06^ |
| ASPH | aspartate beta-hydroxylase | 2.29 | 1.90 X 10^-06^ |
| ITGA2 | integrin, alpha 2 (CD49B, alpha 2 subunit of VLA-2 receptor) | 2.15 | 2.63 X 10^-06^ |
| SRXN1 | sulfiredoxin 1 | 2.31 | 2.63 X 10^-06^ |
| OGT | O-linked N-acetylglucosamine (GlcNAc) transferase | 2.26 | 2.70 X 10^-06^ |
| SELM | Selenoprotein M Precursor (SelM) | 2.24 | 2.75 X 10^-06^ |
| ZNF7 | zinc finger protein 7 | 2.04 | 2.87 X 10^-06^ |
| SLC26A2 | solute carrier family 26 (sulfate transporter), member 2 | 2.27 | 3.39 X 10^-06^ |
| RASEF | RAS and EF-hand domain containing | 2.48 | 3.67 X 10^-06^ |
| C11orf93 | Uncharacterized protein C11orf93 | 2.07 | 4.04 X 10^-06^ |
| HSPB1 | heat shock 27kDa protein 1 | 2.37 | 5.00 X 10^-06^ |
| C1orf85 | Lysosomal protein NCU-G1 Precursor | 2.10 | 5.14 X 10^-06^ |
| TKT | transketolase | 2.54 | 5.56 X 10^-06^ |
| LARP1 | La ribonucleoprotein domain family, member 1 | 2.27 | 5.86 X 10^-06^ |
| ZFP36L2 | zinc finger protein 36, C3H type-like 2 | 2.79 | 6.29 X 10^-06^ |
| LRP11 | low density lipoprotein receptor-related protein 11 | 2.46 | 6.68 X 10^-06^ |
| SRGAP2P1 | Slit-Robo Rho GTPase activating protein 2 pseudogene 1 | 2.72 | 7.73 X 10^-06^ |
| ACSL4 | Acyl-CoA synthetase long-chain family member 4 | 2.40 | 7.74 X 10^-06^ |
| FBXO32 | F-box protein 32 | 2.22 | 8.11 X 10^-06^ |
| DCAF13 | DDB1 and CUL4 associated factor 13 | 2.54 | 9.27 X 10^-06^ |
| LAPTM4B | lysosomal protein transmembrane 4 beta | 2.47 | 1.07 X 10^-05^ |
| VPS13B | vacuolar protein sorting 13 homolog B (yeast) | 2.21 | 1.12 X 10^-05^ |
| RFC3 | replication factor C (activator 1) 3, 38kDa | 2.31 | 1.12 X 10^-05^ |
| DLAT | dihydrolipoamide S-acetyltransferase | 2.19 | 1.12 X 10^-05^ |
| CD24 | CD24 molecule | 2.28 | 1.19 X 10^-05^ |
| TUSC3 | tumor suppressor candidate 3 | 2.22 | 1.26 X 10^-05^ |
| SQLE | squalene epoxidase | 2.84 | 1.29 X 10^-05^ |
| GLUD2 | glutamate dehydrogenase 2 | 2.27 | 1.33 X 10^-05^ |
| SYNJ2 | synaptojanin 2 | 2.05 | 1.38 X 10^-05^ |
| SMC2 | structural maintenance of chromosomes 2 | 2.17 | 1.48 X 10^-05^ |
| NCOA2 | nuclear receptor coactivator 2 | 3.15 | 1.50 X 10^-05^ |
| TMEM38B | transmembrane protein 38B | 2.26 | 1.71 X 10^-05^ |
| ZIC1 | Zic family member 1 (odd-paired homolog, Drosophila) | 2.64 | 2.05 X 10^-05^ |
| APOLD1 | apolipoprotein L domain containing 1 | 2.85 | 2.07 X 10^-05^ |
| SMC4 | structural maintenance of chromosomes 4 | 2.43 | 2.61 X 10^-05^ |
| SRPK2 | SRSF protein kinase 2 | 2.30 | 2.66 X 10^-05^ |
| NIPAL2 | NIPA-like domain containing 2 | 2.26 | 2.71 X 10^-05^ |
| SNORD77 | small nucleolar RNA, C/D box 44 | 2.03 | 2.75 X 10^-05^ |
| PLA2G4C | phospholipase A2, group IVC (cytosolic, calcium-independent) | 2.18 | 2.80 X 10^-05^ |
| NUDCD1 | NudC domain containing 1 | 2.01 | 2.96 X 10^-05^ |
| KIAA0907 | KIAA0907 | 2.20 | 3.02 X 10^-05^ |
| PPP1R9A | protein phosphatase 1, regulatory (inhibitor) subunit 9A | 2.18 | 3.14 X 10^-05^ |
| B3GNT5 | UDP-GlcNAc:betaGal beta-1,3-N-acetylglucosaminyltransferase 5 | 2.90 | 3.23 X 10^-05^ |
| CHMP4C | chromatin modifying protein 4C | 2.29 | 3.59 X 10^-05^ |
| MAGEA1 | melanoma antigen family A, 1 | 2.16 | 3.67 X 10^-05^ |
| FIGNL1 | fidgetin-like 1 | 2.12 | 3.86 X 10^-05^ |
| TMEM65 | transmembrane protein 65 | 2.29 | 4.02 X 10^-05^ |
| HIST2H2AA3 | histone cluster 2, H2aa3 | 2.29 | 4.20 X 10^-05^ |
| CHML | choroideremia-like (Rab escort protein 2) | 2.57 | 4.70 X 10^-05^ |
| UXS1 | UDP-glucuronate decarboxylase 1 | 2.09 | 4.75 X 10^-05^ |
| NXT2 | nuclear transport factor 2-like export factor 2 | 2.31 | 4.91 X 10^-05^ |
| TGIF1 | TGFbeta induced factor homeobox 1 | 2.45 | 5.13 X 10^-05^ |
| TMEM165 | transmembrane protein 165 | 2.20 | 5.21 X 10^-05^ |
| CAMSAP1L1 | calmodulin regulated spectrin-associated protein 1-like 1 | 2.35 | 5.29 X 10^-05^ |
| ILF2 | interleukin enhancer binding factor 2, 45kDa | 2.38 | 5.50 X 10^-05^ |
| LONRF2 | LON peptidase N-terminal domain and ring finger 2 | 2.06 | 5.88 X 10^-05^ |
| UTP14A | UTP14, U3 small nucleolar ribonucleoprotein, homolog A | 2.08 | 6.40 X 10^-05^ |
| PTPRG | protein tyrosine phosphatase, receptor type, G | 2.18 | 6.85 X 10^-05^ |
| TNFAIP3 | tumor necrosis factor, alpha-induced protein 3 | 2.12 | 6.86 X 10^-05^ |
| FMNL2 | formin-like 2 | 2.12 | 8.43 X 10^-05^ |
| EPDR1 | ependymin related protein 1 | 2.34 | 8.81 X 10^-05^ |
| PHLDA2 | pleckstrin homology-like domain, family A, member 2 | 3.12 | 9.22 X 10^-05^ |
| MZT1 | mitotic spindle organizing protein 1 | 2.34 | 9.60x 10^-05^ |
| SULT1C2 | sulfotransferase family, cytosolic, 1C, member 2 | 2.74 | 0.0001 |
| GPD2 | glycerol-3-phosphate dehydrogenase 2 (mitochondrial) | 2.06 | 0.0001 |
| DCTN4 | dynactin 4 (p62) | 2.06 | 0.0001 |
| RASAL2 | RAS protein activator like 2 | 2.07 | 0.0001 |
| PDP1 | pyruvate dehyrogenase phosphatase catalytic subunit 1 | 2.63 | 0.0001 |
| RCOR3 | REST corepressor 3 | 2.06 | 0.0001 |
| CXCL11 | chemokine (C-X-C motif) ligand 11 | 2.09 | 0.0001 |
| C5orf28 | Chromosome 5 open reading frame 28 | 2.17 | 0.0001 |
| RAP2A | RAP2A, member of RAS oncogene family | 2.06 | 0.0002 |
| MED30 | mediator complex subunit 30 | 2.37 | 0.0002 |
| UPF3A | UPF3 regulator of nonsense transcripts homolog A | 2.30 | 0.0002 |
| CASC5 | Cancer suscepibility candidate 5 | 2.05 | 0.0002 |
| MAGEA6 | melanoma antigen family A, 6 | 2.76 | 0.0002 |
| ANXA2 | annexin A2 | 2.03 | 0.0002 |
| ACSL6 | acyl-CoA synthetase long-chain family member 6 | 2.33 | 0.0002 |
| MTERFD1 | MTERF domain containing 1 | 2.02 | 0.0002 |
| LANCL1 | LanC lantibiotic synthetase component C-like 1 | 2.00 | 0.0003 |
| CST1 | cystatin SN | 2.29 | 0.0003 |
| HS2ST1 | heparan sulfate 2-O-sulfotransferase 1 | 2.19 | 0.0003 |
| MERTK | c-mer proto-oncogene tyrosine kinase | 2.05 | 0.0003 |
| NUP37 | nucleoporin 37kDa | 2.00 | 0.0003 |
| SLC38A1 | solute carrier family 38, member 1 | 2.37 | 0.0003 |
| UBE2Q2 | ubiquitin-conjugating enzyme E2Q family member 2 | 2.52 | 0.0003 |
| VPS13C | Vacuolar protein sorting 13 homolog C | 2.17 | 0.0004 |
| FEM1C | fem-1 homolog c (C. elegans) | 2.01 | 0.0004 |
| LIN28B | lin-28 homolog B (C. elegans) | 2.27 | 0.0004 |
| ZHX1 | zinc fingers and homeoboxes 1 | 2.03 | 0.0005 |
| DOCK7 | dedicator of cytokinesis 7 | 2.13 | 0.0005 |
| PLCB1 | phospholipase C, beta 1 (phosphoinositide-specific) | 2.11 | 0.0005 |
| RBM15 | RNA binding motif protein 15 | 2.07 | 0.0005 |
| PIR | pirin (iron-binding nuclear protein) | 2.03 | 0.0005 |
| ME1 | malic enzyme 1, NADP(+)-dependent, cytosolic | 2.49 | 0.0006 |
| DPP4 | dipeptidyl-peptidase 4 | 2.33 | 0.0006 |
| PTBP2 | polypyrimidine tract binding protein 2 | 2.02 | 0.0006 |
| CLIP4 | CAP-GLY domain containing linker protein family member 4 | 2.07 | 0.0006 |
| PHF6 | PHD finger protein 6 | 2.03 | 0.0007 |
| CRTAP | cartilage associated protein | 2.22 | 0.0007 |
| TRNT1 | tRNA nucleotidyl transferase, CCA-adding, 1 | 2.09 | 0.0007 |
| EIF3B | eukaryotic translation initiation factor 3, subunit B | 2.12 | 0.0007 |
| ERAP2 | endoplasmic reticulum aminopeptidase 2 | 2.54 | 0.0008 |
| HIST1H2AC | histone cluster 1, H2ac | 2.40 | 0.0008 |
| CTSC | cathepsin C | 2.66 | 0.0008 |
| PPP1R2 | protein phosphatase 1, regulatory (inhibitor) subunit 2 pseudogene 3 | 2.27 | 0.0008 |
| HIST1H1C | histone cluster 1, H1c | 2.48 | 0.0009 |
| RGS1 | regulator of G-protein signaling 1 | 2.71 | 0.0009 |
| ZBTB41 | Zinc finger and BTB domain containing 41 | 2.32 | 0.0009 |
| PDE4DIP | Phosphodiesterase 4D interacting protein | 2.08 | 0.001 |
| QSER1 | glutamine and serine rich 1 | 2.07 | 0.0013 |
| DERL1 | Der1-like domain family, member 1 | 2.19 | 0.0013 |
| LNPEP | leucyl/cystinyl aminopeptidase | 2.24 | 0.0014 |
| PGC | progastricsin (pepsinogen C) | 2.11 | 0.0014 |
| CHD2 | Chromosome helicase DNA binding protein 2 | 2.12 | 0.0015 |
| MYC | v-myc myelocytomatosis viral oncogene homolog | 3.06 | 0.0015 |
| PSPH | phosphoserine phosphatase | 3.88 | 0.0015 |
| TCERG1 | transcription elongation regulator 1 | 2.31 | 0.0015 |
| BIRC3 | baculoviral IAP repeat-containing 3 | 2.38 | 0.0016 |
| ANP32E | Acidic (leucine rich) nuclear phosphoprotein 32 family member E | 2.14 | 0.0016 |
| GLUL | glutamate-ammonia ligase | 2.18 | 0.0017 |
| CAPRIN1 | cell cycle associated protein 1 | 2.00 | 0.0018 |
| BAG2 | BCL2-associated athanogene 2 | 2.22 | 0.0018 |
| JMY | junction mediating and regulatory protein, p53 cofactor | 2.07 | 0.0019 |
| DPH3 | DPH3, KTI11 homolog | 2.16 | 0.0021 |
| YTHDF3 | YTH domain family, member 3 | 2.50 | 0.0022 |
| C14orf147 | Small subunit of serine palmitoyltransferase A (ssSPTa) | 2.40 | 0.0022 |
| ASPH | aspartate beta-hydroxylase | 2.13 | 0.0023 |
| HMGCS1 | 3-hydroxy-3-methylglutaryl-CoA synthase 1 | 2.46 | 0.0023 |
| AKR1C1 | Aldoketoreductase family 1, member C1 | 2.12 | 0.0023 |
| CSE1L | CSE1 chromosome segregation 1-like | 2.11 | 0.0024 |
| SEPP1 | selenoprotein P, plasma, 1 | 2.38 | 0.0028 |
| HSP90AB1 | heat shock protein 90kDa alpha (cytosolic), class B member 1 | 2.27 | 0.0033 |
| ARPP19 | cAMP-regulated phosphoprotein, 19kDa | 2.11 | 0.0035 |
| JAG1 | jagged 1 | 2.19 | 0.0040 |
| ADAM9 | ADAM metallopeptidase domain 9 | 2.25 | 0.0043 |
| ITGAV | integrin, alpha V (vitronectin receptor, alpha polypeptide, antigen CD51) | 2.20 | 0.0044 |
| POLR2K | polymerase (RNA) II (DNA directed) polypeptide K, 7.0kDa | 2.08 | 0.0044 |
| ZBTB38 | zinc finger and BTB domain containing 38 | 2.17 | 0.0049 |
| MRPL13 | mitochondrial ribosomal protein L13 | 2.01 | 0.005 |
| UGT1A8 | UDP glucuronosyltransferase 1 family, polypeptide A8 | 2.15 | 0.005 |
| NEDD9 | neural precursor cell expressed, developmentally down-regulated 9 | 2.38 | 0.0064 |
| NEAT1 | Nuclear paraspeckle assembly transport 1 (non protein coding) | 2.00 | 0.0066 |
| ZNF207 | zinc finger protein 207 | 2.07 | 0.0070 |
| IL32 | interleukin 32 | 2.33 | 0.0070 |
| SLC25A32 | solute carrier family 25, member 32 | 2.10 | 0.0071 |
| PHACTR2 | phosphatase and actin regulator 2 | 2.16 | 0.0072 |
| KLF6 | Kruppel-like factor 6 | 2.41 | 0.0072 |
| CXCR7 | chemokine (C-X-C motif) receptor 7 | 2.12 | 0.0074 |
| CD55 | CD55 molecule, decay accelerating factor for complement (Cromer blood group) | 2.19 | 0.0079 |
| GPNMB | glycoprotein (transmembrane) nmb | 2.06 | 0.008 |
| TBL1XR1 | transducin (beta)-like 1 X-linked receptor 1 | 2.17 | 0.01 |
| ZFAND5 | zinc finger, AN1-type domain 5 | 2.22 | 0.0144 |
| IFNGR1 | interferon gamma receptor 1 | 2.20 | 0.015 |
| PDK4 | pyruvate dehydrogenase kinase, isozyme 4 | 2.11 | 0.018 |
| DHRS2 | dehydrogenase/reductase (SDR family) member 2 | 2.32 | 0.032 |

**Table S3** Genes with > 2-fold change in expression unique to HBV-related HCC compared to normal liver.
