# Supplementary material for: Global Gene Expression Profiling Reveals SPINK1 as a Potential Hepatocellular Carcinoma Marker
Source: PLoS One. 2013 Mar 18;8(3):e59459. doi: 10.1371/journal.pone.0059459 (PMC3601070; doi:10.1371/journal.pone.0059459)
Supplement: Table S4 — Gene ontology analysis of differential gene expression, showing top 10 significantly enriched terms comparing HBV with HBV-HCC and comparing HCV with HCV-HCC. (DOCX) [file pone.0059459.s004.docx]

**Table S4** Gene ontology analysis of differential gene expression, showing top 10 significantly enriched terms comparing HBV with HBV-HCC and comparing HCV with HCV-HCC. p-value according to Fishers exact test.

|  | **GO term** | **p-value** |
| --- | --- | --- |
| HBVvs HBV-HCC – Biological processes | peptide cross-linking | 2.7 x 10^-06^ |
|  | sulfur compound metabolic process | 9.80 x 10^-06^ |
|  | glycosaminoglycan metabolic process | 9.80 x 10^-06^ |
|  | regulation of organ morphogenesis | 2.90 x 10^-05^ |
|  | humoral immune response | 5.40 x 10^-05^ |
|  | developmental growth involved in morphogenesis | 0.00011 |
|  | negative regulation of peptidase activit... | 0.00017 |
|  | bone morphogenesis | 0.00017 |
|  | regulation of morphogenesis of a branchi... | 0.00017 |
|  | placenta development | 0.00026 |
| HBV vs. HBV-HCC  Molecular function | extracellular matrix binding | 4.30 x 10^-07^ |
|  | glycosaminoglycan binding | 5.20 x 10^-10^ |
|  | collagen binding | 2.00 x 10^-06^ |
|  | heparin binding | 4.90 x 10^-05^ |
|  | monooxygenase activity | 0.00013 |
|  | extracellular matrix structural constitu... | 0.00074 |
|  | oxidoreductase activity, acting on paire... | 0.00074 |
|  | G-protein-coupled receptor binding | 0.00125 |
|  | transmembrane receptor activity | 0.00181 |
|  | endopeptidase regulator activity | 0.00252 |
| HCV vs. HCV-HCC  Biological processes | oxidation reduction | 1.10 x 10^-12^ |
|  | drug metabolic process | 1.10 x 10^-12^ |
|  | immune response | 3.20 x 10^-13^ |
|  | tryptophan catabolic process to kynureni... | 4.70 x 10^-06^ |
|  | peptide cross-linking via chondroitin 4-... | 6.70 x 10^-06^ |
|  | retinoic acid metabolic process | 9.40 x 10^-06^ |
|  | complement activation, alternative pathw... | 1.30 x 10^-05^ |
|  | gluconeogenesis | 1.60 x 10^-05^ |
|  | parturition | 1.70 x 10^-05^ |
| HCV vs. HCV-HCC  Molecular function | oxygen binding | 1.90 x 10^-09^ |
|  | heme binding | 6.30 x 10^-17^ |
|  | electron carrier activity | 1.30 x 10^-14^ |
|  | aromatase activity | 1.10 x 10^-13^ |
|  | chemokine activity | 5.50 x 10^-09^ |
|  | antigen binding | 1.50 x 10^-08^ |
|  | cadmium ion binding | 2.80 x 10^-07^ |
|  | retinal binding | 6.70 x 10^-06^ |
|  | oxidoreductase activity, acting on paire... | 8.50 x 10^-06^ |
|  | prostaglandin E receptor activity | 9.30 x 10^-06^ |
